# A Computational-Experimental Approach to Unravel the Excited State Landscape in Heavy-Atom Free BODIPY-Related Dyes

**DOI:** 10.3390/molecules27154683

**Published:** 2022-07-22

**Authors:** Esther Rebollar, Jorge Bañuelos, Santiago de la Moya, Julien Eng, Thomas Penfold, Inmaculada Garcia-Moreno

**Affiliations:** 1 Departamento Química-Física de Materiales, Instituto de Química Física “Rocasolano”, CSIC, Serrano 119, 28006 Madrid, Spain; e.rebollar@csic.es; 2 Departamento de Química Física, Universidad del País Vasco-EHU, Apartado 644, 48080 Bilbao, Spain; 3 Departamento Química Orgánica, Facultad de Ciencias Químicas, Universidad Complutense de Madrid, Ciudad Universitaria s/n, 28040 Madrid, Spain; santmoya@ucm.es; 4 Chemistry Department, School of Natural and Environmental Sciences, Newcastle University, Newcastle Upon-Tyne NE1 7RU, UK; julien.eng@newcastle.ac.uk (J.E.); tom.penfold@newcastle.ac.uk (T.P.)

**Keywords:** BODIPY dyes, delayed fluorescence, reverse intersystem crossing, CC2 calculations, laser spectroscopy

## Abstract

We performed a time-gated laser-spectroscopy study in a set of heavy-atom free single BODIPY fluorophores, supported by accurate, excited-state computational simulations of the key low-lying excited states in these chromophores. Despite the strong fluorescence of these emitters, we observed a significant fraction of time-delayed (microseconds scale) emission associated with processes that involved passage through the triplet manifold. The accuracy of the predictions of the energy arrangement and electronic nature of the low-lying singlet and triplet excited states meant that an unambiguous assignment of the main deactivation pathways, including thermally activated delayed fluorescence and/or room temperature phosphorescence, was possible. The observation of triplet state formation indicates a breakthrough in the “classic” interpretation of the photophysical properties of the renowned BODIPY and its derivatives.

## 1. Introduction

Purely organic materials, which harvest triplet excitons through thermally activated delayed fluorescence (TADF) and/or room-temperature phosphorescence (RTP), have attracted extensive interest because of their potential applications in optics, electronics, biology and so forth [1,2,3,4,5,6,7,8]. However, triplet exciton generation in heavy-atom free organic materials is often challenging, due to ineffective spin-orbit coupling. Different and promising methods for triggering the otherwise restricted intersystem crossing (ISC) have been proved in recent years, such as exciton coupling, spin-converter, photoinduced electron transfer, symmetric breaking charge recombination and radical-enhanced ISC [9,10,11,12]. However, despite the research efforts to develop purely-organic TADF and RTP materials, most of them are based on synthetically demanding molecular structures requiring complex and lengthy synthesis [7,13,14,15,16,17,18,19,20]. Consequently, long-lived room-temperature emissions from heavy-atom free organic materials in aerated solutions are still rare, and are highly sought after. 

**Scheme 1:**
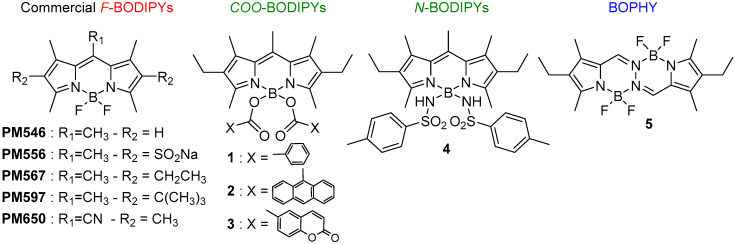
Molecular structures of the tested BODIPY and BOPHY dyes.

The high chemical feasibility of the BODIPY (boron dipyrromethene) chromophore makes it a unique framework to develop long-lived emitting materials [21]. RTP and TADF have only been recorded from heavy-atom functionalized BODIPY dyes, or from heavy-atom free BODIPY chromophores built into complex architectures, elaborated derivatives and multichromophoric arrays, among others, as advanced approaches to triggering ISC effectiveness for promoting triplet harvesting [9,10,11,21,22,23,24]. In this context, long-lived emissions from commercially available monochromophoric BODIPYs have not been considered due to their assumed lack of effective ISC [25,26,27]. Nonetheless, the versatility and performance of laser radiation systems, coupled with high-gain detection equipment, allowed us to record such unexpected long-lived RTP and TADF from aerated solutions of commercial BODIPYs (Scheme 1) [28]. Such unnoticed phenomenon spans the “well-known” photophysics of one of the most studied dye families so far, allowing for a well-grounded comparison with quantum mechanical methods in predicting the electronic nature of their low-lying excited states.

Several studies have been conducted using theoretical simulations of the electronically-excited-state properties of BODIPYs [28,29,30,31,32,33,34], most of them focused on the quantification of the S_1_/S_0_ and T_1_/S_1_ energy gaps, which is critical to understanding their photophysical behavior upon excitation. However, to the best of our knowledge, none of the conclusions were supported by an accurate correlation with the experimental measurements of prompt and delayed emissions. In this regard, the present work envisioned an innovative joint experimental-theoretical approach to provide convincing evidence for an unambiguous TADF and RTP assignment through a quantitative estimation of the S_1_, T_1_ and T_2_ energies, assessing the performance of the current quantum mechanical methods in predicting the energy arrangement and electronic nature of the low-lying excited-states of BODIPYs. In addition, the feasibility of tuning triplet harvesting from these commercial and structurally simple BODIPY dyes to related at-boron-substituted and BODIPY-like (BOPHY) [35,36] derivatives (Scheme 1) was also analyzed, leading to deeper insights into the underlying factors that control the elusive relationships between structure and TADF/RTP properties, the latter being key in the rational design of smarter long-lived emitting materials for advanced applications. 

## 2. Results and Discussion

### 2.1. Commercial BODIPY Dyes

**Figure 1:**
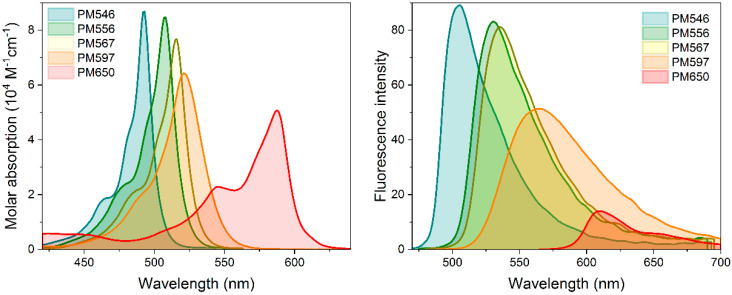
Absorption (scaled by the molar absorption) and steady-state fluorescence (scaled by the fluorescence efficiency) spectra of the commercial BODIPYs in diluted solutions (2 μM) of acetonitrile.

Alkylated BODIPYs PM546 and PM567, and PM556 bearing water-solubilizing 2,6-sulfonate groups, displayed strong absorption and steady-state fluorescence bands, with efficiencies ranging around 80–90% (Figure 1 and Table S1 in Supplementary Materials) [27]. In contrast, the attachment of branched 2,6-*tert*-butyls in the alkylated PM597 enhanced the Stokes shift and decreased the fluorescence efficiency by half, owing to the steric-hindrance-induced bending of the chromophore geometry (Figure 1 and Table S1 in Supplementary Materials) [27]. In all of them, the solvent polarity induced low negative solvatochromism and a modest decrease of the absorption and fluorescence probability. The exception to this rule is PM650 bearing an 8-cyano moiety, where the red-shifted emission (Figure 1) was sensitive to the solvent properties. Thus, the strong electron withdrawing cyano group was able to photoinduce a “dark” intramolecular charge transfer (ICT) process, which quenched the emission from the locally excited (LE) state. This non-radiative channel was mainly enabled in polar solvents, where the charge separation of the ICT is further stabilized, and the ICT became the low-lying state and a sink for the excited electrons (Table S1 in Supplementary Materials) [27]. Time-resolved spectroscopy provided monoexponential decays with lifetimes in the nanoseconds scale, whose evolution matched that of the fluorescence quantum yield (a decline of the prompt fluorescence efficiency by solvent effect or molecular structure implied a faster lifetime, Table S1 in Supplementary Materials). None of these dyes showed phosphorescence emission at low temperatures (77 K) or singlet oxygen emission at 1270 nm, either in degassed or aerated solutions, suggesting that the triplet state population was rather low in these fluorescent dyes under mild irradiation conditions. Indeed, previous computational studies revealed that the probability of intersystem crossing is low, owing to the high singlet-triplet energy barrier and mainly low spin-orbit coupling (SOC) [29].

**Figure 2:**
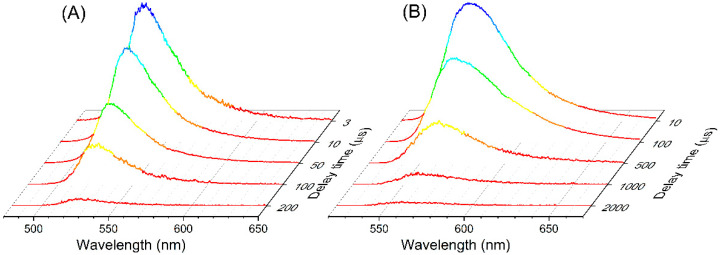
Time-dependent fluorescence emission spectra of PM546 in ethyl acetate (**A**) and PM556 in DMSO (**B**) measured under ambient conditions. Optically matched solutions at 532 nm were used.

Surprisingly, under exposure to intense laser pulses at 532 nm, all the selected commercial BODIPY dyes (PM546, PM556, PM567, PM597 and PM650, see Scheme 1) in ethyl acetate solution exhibited emission at delay times up to 200 μs with a spectral profile similar to its prompt laser-induced fluorescence. These emissions, registered using, laser-induced time-gated spectroscopy, are red-shifted with respect to those recorded by means of steady state spectroscopy, owing to the different optical density of the samples (0.1 mM and 2 μM, respectively, both in cuvettes of 1 cm; see experimental section), and the ensuing reabsorption/reemission phenomena in concentrated media. This delayed spectral signature remained unchanged over the exposure time, except for a steady decrease in its intensity as the delay time increases (e.g., see Figure 2 for PM546 and PM556). The recorded time-delayed emission is independent on increasing solvent polarity from toluene (Rohrschneider’s polarity parameter, P = 2.3) to ethyl acetate (P = 4.3), and then to acetonitrile (P = 6.2). In this regard, delayed emission from PM556 with sulfonate groups at the 2 and 6 positions of the BODIPY core (Figure 2) was only recorded in DMSO due to its low solubility and fast laser-induced photodegradation in other polar solvents. The higher viscosity of DMSO allowed a delayed emission to be recorded, matching its prompt fluorescence beyond 2 ms after the incoming laser radiation. This is an astonishing behavior, considering that it was recorded in an aerated solution at room temperature. Note that such a time-gated emission was not detected under the standard irradiation conditions used to record the photophysical properties.

Considering that prompt fluorescence involves lifetimes of around 4–6 ns, delayed emission with lifetimes longer than 100 μs must unequivocally imply the involvement of long-lived triplet excited states. Moreover, the intensity of the recorded delayed emission increased linearly by increasing the laser pulse energy (Figure S1 in Supplementary Materials), following therefore the expected dependence of one-photon processes such as TADF. At this point, it could be hypothesized that triplet-triplet annihilation upconversion (TTA-UC) lies in the origin of this prompt-like delayed fluorescence. However, an efficient TTA-UC requires, among other factors, a high enough concentration of annihilating species (higher than 5 × 10^−4^ M in referable organic dyes) [37], which is virtually impossible to reach under the experimental conditions selected here to record delayed emissions. More specifically, the low dye concentration (<1 × 10^−4^ M), as well as the absence of other chromophores acting as triplet acceptors (annihilators) and the presence of oxygen effectively quenching triplet states drastically reduced the concentration of T_1_ excitons, and, consequently, the probability of the bimolecular interactions required for its effective annihilation.

**Figure 3:**
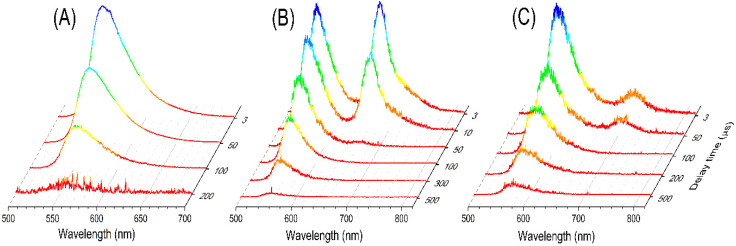
Time-dependent emission spectra of PM567 in ethyl acetate (**A**) and in *n*-propyl iodide (**B**), and PM597 in *n*-propyl iodide (**C**) recorded under ambient conditions after laser photo-excitation at 532 nm. Optically matched solutions were used.

More interestingly, PM567 and PM597 dissolved in a halogenated solvent like *n*-propyl iodide exhibited the growth of a dual delayed emission, which involves a new, minority long-wavelength band centered at 680 nm and 710 nm, respectively (Figure 3). While the observed short-wavelength emissions matched the respective prompt fluorescence, and consequently it was possible to assign them to TADF, the long-wavelength emissions should be tentatively assigned to RTP, so that the energy of the T_1_ excited state can be assessed experimentally. Therefore, in this simple BODIPY system, it was possible to harvest triplet states using rISC, giving rise to TADF, and also decay radiatively to the ground state, giving rise to RTP. It must be noted that the detection of TADF effectively competing with RTP detection is not merely related to the probability of the rISC (from T_2_ to S_1_) and IC (from T_2_ to T_1_) processes involved, the latter being much more probable, but to the relative efficiency of the subsequent radiative de-excitation processes. Thus, the radiative S_1_-to-S_0_ deactivation involved in the TADF emission is much more efficient than the T_1_-to-S_0_ one involved in the RTP, the latter also being more efficiently quenched by oxygen. All these factors taken together could account for the recorded TADF/RTP ratio and the faster disappearance of the RTP emission, which lived less than the corresponding TADF (Figure 3).

**Figure 4:**
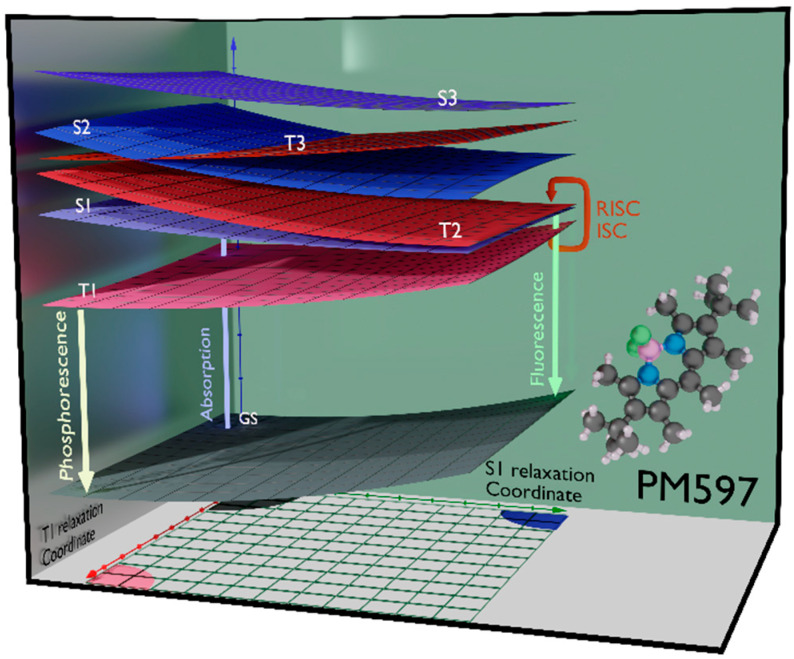
Schematic potential energy surface of representative PM597 interpolated along the T_1_ and S_1_ relaxation coordinates. The interpolation is performed assuming a harmonic potential between the ground-state optimized geometry and either the S_1_ or T_1_ minimum geometry. Only the energies of the electronic states at the ground state, T_1_ and S_1_ optimized geometries are computed. Crucially, the close proximity of the S_1_ and T_2_ states at the S_1_ minimum is clearly visible at the S_1_ minimum energy geometry.

These spectral interpretations were supported by the calculation of the electronically excited states of BODIPYs, which is essential to predicting the thermodynamic viability of the deactivation pathways promoted upon photo-excitation. Owing to the challenges of TD-DFT for an accurate description of the excited states of BODIPYs [31,38], singlet and triplet excited states were computed here with the LR-CC2 method. As stated above, the commercial BODIPYs exhibited an extremely long lifetime inconsistent with prompt fluorescence, suggesting the involvement of the triplet manifold. Thus, for PM546 at ground-state geometry, the energy gap between the S_1_ and T_1_ states was 0.8 eV, which is too large for efficient ISC. However, the T_2_ state laid only 0.35 eV above the S_1_. At the relaxed S_1_ geometry, from where fluorescence occurs, the S_1_ was at 2.36 eV, matching the observed fluorescence very well. Here, the T_2_ state dropped below the S_1_ with a gap of 0.12 eV. The T_1_ was a further 0.2 eV below this at 2.04 eV (Table S2 in Supplementary Materials). This smaller energy gap might promote some ISC, generating an equilibrium between the S_1_ and T_2_ states. This mechanism would preclude relaxation down to the T_1_ state, as this is much lower in energy, and not be able to return. A similar picture emerges from PM567 (Table S2 in Supplementary Materials), where the S_1_ and T_2_ states coincide, and a larger gap was observed to the T_1_ state. As shown in Figure 2, using *n*-propyl iodide to exploit the heavy-atom effect and promote ISC, a new low-energy emission band emerged at 680 nm (1.82 eV). This matches the energy of the T_1_ state calculated at the relaxed T_1_ geometry exactly (Table S1 in Supporting Information). Moreover, in such a halogenated solvent, PM597 also exhibited a dual emission at 575 and 710 nm (Figure 2), close to the calculated fluorescence and phosphorescence energies of 610 and 704 nm, respectively. Again, the T_2_ state was close in energy to the S_1_ state, which would promote ISC and an equilibrium between the low-lying singlet and triplet states. Figure 4 illustrates the lowering of the S_1_–T_2_ energy gap upon the relaxation of both states, thus enabling, from a thermodynamic point of view, the back-and-forth movement (ISC/rISC) of excited electrons between both states. 

### 2.2. At-Boron-Functionalized BODIPY Dye and Related Chromophores 

**Figure 5:**
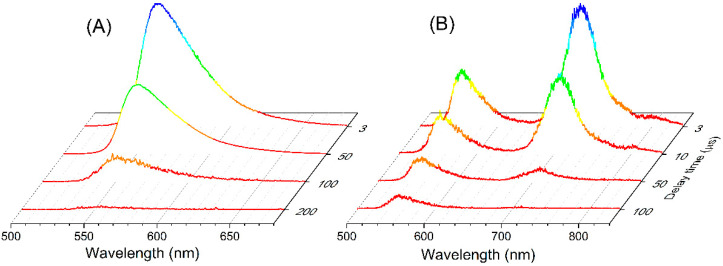
Time-dependent emission spectra of *COO*-BODIPY **2** in ethyl acetate (**A**) and *n*-propyl iodide (**B**) measured under ambient conditions after laser photo-excitation at 532 nm. Optically matched solutions were used.

Time-delayed emissions were also recorded from BODIPYs functionalized at their boron atom, such as *COO-* and *N*-BODIPYs (see **1**–**3** and **4**, respectively, in Scheme 1), the former involving two acyloxyl moieties grafted at the boron center [39,40], the latter bearing 4-methylbenzenosulfonamide functional groups replacing the fluorine atoms [41]. In addition, the BOPHY family (BODIPY-like fluorophores which have a central hydrazine-based spacer in the chelating ligand) was also tested (see dye **5** in Scheme 1) [42]. Despite the electrophilic character at the boron center being altered through the substitution of fluorine atoms by acyloxyl (**1**–**3**) and sulfonylamino (**4**) moieties, a time-delayed emission was recorded from all of these fluorescent, at-boron-functionalized BODIPY dyes (**1**–**4**), as well as from fluorescent BOPHY **5** (Figure 5 and Figure S2 in Supplementary Materials). These long-lived emissions followed a similar pattern to that recorded from parent PM567 (Figure 3). Although the large size of these at-boron-functionalized BODIPYs and BOPHY prevented the accurate and advanced excited-state calculation level previously applied to the commercial BODIPYs, the similarity of the photophysical behavior meant it could be assigned to the corresponding delayed fluorescence. Interestingly, coumarin-involving **3** revealed the viability of an intramolecular excitation energy transfer (EET) process to trigger the delayed fluorescence of the PM567 moiety upon photo-excitation at 355 nm (Figure S3 in Supplementary Materials). In this regard, the EET sustained by **3** became a non-radiative IC process which is more effective in harvesting excitation-light into triplet excitons than the direct UV-excitation of the high-lying singlet excited states of PM567 that led to a nearly undetectable delayed fluorescence emission (see Figure S3 in Supplementary Materials). Likewise, dual time-delayed emissions were also recorded in *n*-propyl iodide. The behavior of **2** was especially striking, where the long-wavelength emission tentatively assigned to RTP appeared as the main band, with RTP/TADF intensity ratio up to 2.5 at 3 μs delay-time (Figure 5). In fact, the phosphorescence features of **2** might arise from the feasibility of the involved anthracene grafted at-boron moiety to intercalate its T_1_ state [43], just below the T_2_ of PM567, promoting triplet-triplet IC, and thus playing a fundamental role in enhancing the population of the low-lying BODIPY triplet state (T_1_) and its subsequent radiative decay to the ground state (S_0_). 

### 2.3. Charge Transfer Role in the Delayed Emission: The Example of PM650 

**Figure 6:**
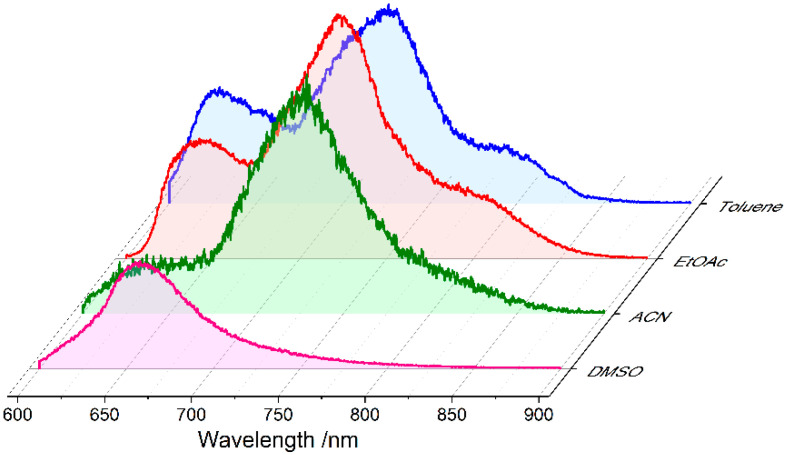
Dependence on the solvent polarity of the time-resolved emission spectra of PM650 recorded at 10 μs delay time after laser photo-excitation at 532 nm in aerated solution at room temperature.

An exception to the behavior observed so far came from PM650 (see Scheme 1), whose emission was mediated by an ICT process induced by the electron-withdrawing cyano group involve, as mentioned in Section 2.1 [44]. Consequently, its photophysical properties were strongly dependent on the polarity of the surrounding environment. Thus, an increase in solvent polarity did not alter the spectral profile of the prompt fluorescence (Figure 1), but implied a progressive decrease in the fluorescence efficiency, becoming almost non-fluorescent in strongly polar solvents (Table S1 in Supplementary Materials). Moreover, the maximum fluorescence wavelength was red-shifted and the lifetime shortened [44]. Coincidently, its time-delayed emission was also sensitive to polarity (Figure 6 and Figure S4 in Supplementary Materials). Thus, in solvents of low-medium polarity such as toluene, ethyl acetate and acetonitrile, the delayed spectrum showed two discernible emission bands: a prompt-like fluorescence emission centered at 640 nm, and a broad, red-shifted band at ca. 730 nm, with a relative intensity ratio decreasing from 0.7 to 0.2 on going from toluene to acetonitrile (Figure 6). In addition, the lifetime of the delayed emission was also strongly affected by the polarity change, decreasing from 200 μs in toluene, to 80 μs in ethyl acetate, and then to 50 μs in acetonitrile, this being the shortest delayed emission recorded from the commercial BODIPYs studied here (Figure S4 in Supplementary Materials). LR-CC2-based calculations at the relaxed S_1_ geometry of PM650 predicted an emission energy at 1.72 eV (Table S2 in Supplementary Materials), matching the prominent emission recorded at 730 nm (Figure 6). This S_1_ with CT-character lays below the locally excited (LE) state with ππ* character on the BODIPY core. Moreover, the relaxed T_1_ geometry places the phosphorescence energy at 1.43 eV (Table S2 in Supplementary Materials), matching the emission recorded at 830 nm as a shoulder in the CT-delayed emission band (Figure 6). We propose that ICT induced by the cyano moiety reinforces ISC [23,45] enabling the recording of a dim phosphorescence simultaneously to its delayed emission under laser irradiation. Solvent-driven ICT-stabilization favors the charge separation, causing its own fluorescence as well as the ensuing ICT-enabled phosphorescence emission to vanish, so that just the weak emission from the LE remains (Figure 6).

## 3. Materials and Methods

### 3.1. Materials

Commercial BODIPY dyes (PM546, PM567, PM597, PM605 and PM650 in Scheme 1) were purchased from Exciton Luxottica (Lockbourne, OH, USA) (laser grade) and were used as received without further purification. The at-boron modified *COO*- (**1**–**3**) and *N*-BODIPYs (**4**), as well as the BOPHY (**5**), of Scheme 1, were synthetized by us, and the corresponding synthetic details and characterization were previously reported [39,40,41,42].

### 3.2. Steady-State and Time-Resolved Spectroscopy

Absorption and fluorescence spectra were registered from diluted solutions (micromolar) on a Varian model CARY 4E spectrophotometer (Agilent, Santa Clara, CA, USA) and an spectrofluorometer (model FLSP920, Edinburgh Instruments, Livingston, UK), using quartz cuvettes with an optical path length of 1 cm. These diluted solutions for photophysical characterization were produced from concentrated solutions in acetone (milimolar). To this aim, a given amount of the stock solution was evaporated under vacuum and the residue redissolved in the desired solvent. Fluorescence quantum yields (φ) were determined by the optically dilute relative method, using standards (purchased from Exciton) as references (fluorescein, φ^r^ = 0.97 in ethanol, and cresyl violet, φ^r^ = 0.54 in methanol). Low-temperature fluorescence spectra were recorded with a liquid-nitrogen-cooled cryostat coupled to the aforementioned spectrofluorometer (Edinburgh Instruments, Livingston, UK). This technique also uses an InGaAs detector suitable for recording NIR emissions, such as those from singlet oxygen. The spectrofluorimeter was equipped with a time-correlated single-photon counter. The radiative decay curves were monitored at the maximum fluorescence wavelength, after excitation with a picosecond-pulsed wavelength-tunable Fianium laser. Detection was carried out with a microchannel plate detector (Hamamatsu C4878, Edinburgh Instruments, Livingston, UK). The fluorescence lifetime (τ) was obtained from the slope of the exponential fit of the decay curve, after the deconvolution of the instrumental response signal (recorded with a ludox scattering suspension) by means of an iterative method. The accuracy of the fit was controlled by chi-square statistical parameters and the analysis of the distribution of the residuals. 

### 3.3. Laser Spectroscopy 

BODIPYs and BOPHYs were transversally excited at 355 nm and/or 532 nm by using the third and mainly second harmonic of a Nd:YAG laser (LS-2147, LOTIS TII, Minsk, Belarus) working at 10 Hz repetition rate. Measurements were carried out at room temperature in aerated solutions contained in 1-cm optical-path rectangular quartz cells. Delayed emissions were recorded from dye concentrations lower than 0.1 mM and at energy fluences lower than 10 mJ/cm^2^, which are experimental conditions assured to be well below the threshold for onsetting the laser action. 

The time-gated emission upon laser photoexcitation was analyzed perpendicularly to the input radiation and was displayed on a spectrograph (Kymera 193i-A, Andor Technologies, Belfast, UK) coupled to an intensified CCD camera (iStar, Andor Technologies). The camera enabled gate widths ranging from nanoseconds to seconds, whose opening can be delayed in a controlled way with respect to the incoming pump laser pulse. Neither long-pass-filters nor band-pass filters were used to remove the excitation laser, since these filters exhibited their own fluorescence and/or phosphorescence emission, which could lead to the experimental results being misunderstood. Each spectrum is the average of at least 200 scans, recorded with a gate time of 50 μs. This experimental set-up enabled the projected measurements to be carried out even under adverse conditions such as aerated and diluted solutions at room temperature, but meant that the efficiency of the delayed emission was not accurately determined.

### 3.4. Computational Simulations

Ground-state and excited-state structural optimization were performed with the QChem package (Q-Chem, Inc., Pleasanton, CA, USA) [46], along with the PBE0 [47,48] functional and the def2-TZVP [49] all-electron basis set. The singlet and triplet excited-state optimized geometries were computed using the advanced linear-response coupled-cluster (LR-CC2) method, using the same basis set, as implemented within the Turbomole [50] quantum chemistry package (TURBOMOLE GmbH, Karlsruhe, Germany). All energies are reported relative to the ground-state energy at the ground-state optimized geometry.

## 4. Conclusions

In summary, the experimental-theoretical characterization of heavy-atom free BODIPY-related dyes conducted demonstrated the feasibility of ISC as a deactivation pathway, despite a low spin-orbit coupling. Accurate excited-state simulations predicted a relaxed T_2_ state accessible from the low-lying S_1_ as key to sustaining recorded time-delayed emission such as TADF through a rISC process and/or RTP through intersystem crossing and ensuing internal conversion down to T_1_ state, depending on the physicochemical properties of the solvent (polarity or halogenation) and the BODIPY molecular structure. In fact, all the dyes tested showed delayed-fluorescence lasting hundreds of microseconds under laser excitation. In particular, PM650 yielded simultaneous TADF/RTP emissions without any additive to promote ISC, owing to its ability to sustain effective ICT, which mediates in the triplet state population. These results indicate a breakthrough in the classic interpretation of the photophysical properties of the renowned BODIPY dyes. Furthermore, time-gated laser spectroscopy, endorsed by excited state calculations, would establish a new design method for using BODIPY-related dyes for photonic applications requiring long-lived emissions.

## Data Availability

The data presented in this study are available on request from the corresponding author.

## Supplementary Materials

The following are available online at https://www.mdpi.com/article/10.3390/molecules27154683/s1; Table S1: Photophysical properties of the commercial BODIPYs in diluted solutions (2 mM) of representative solvents; Table S2: LR-CC2 results of the low-lying excited states and oscillator strengths (in brackets) of PM546, PM567, PM597 and PM650 at the ground, S_1_ and T_1_ optimised geometries; Figure S1: Intensity of the recorded delayed emission as a function of the laser pulse energy; Figure S2: Time-dependent fluorescence emission spectra of *COO*-BODIPY **3** upon laser photo-excitation at 355 nm (A), and *N*-BODIPY **4** (B) and BOPHY **5** (C) after laser photo-excitation at 532 nm, in ethyl acetate aerated solution at room temperature; Figure S3: Time-resolved emission spectra at different delay times (in μs) after laser excitation at 355 nm of PM546 (left) and PM597 (right) in aerated ethyl acetate solution at room temperature; Figure S4: Comparison of the time-resolved emission spectra of PM650 in different solvents (toluene (A), ethyl acetate (B), acetonitrile (C) and DMSO (D)) upon laser photoexcitation at 532 nm. The measurements were carried out in aerated solutions at room temperature.

## Sample Availability

Samples of all compounds are available from the authors.
