# Supplementary material for: A Computational-Experimental Approach to Unravel the Excited State Landscape in Heavy-Atom Free BODIPY-Related Dyes
Source: Molecules. 2022 Jul 22;27(15):4683. doi: 10.3390/molecules27154683 (PMC9330419; doi:10.3390/molecules27154683)
Supplement: Supplementary file 1 [file molecules-27-04683-s001.zip › molecules-1798920-supplementary.pdf]

Supplementary Materials

A Computational-Experimental Approach to Unravel the Excited State Landscape in Heavy-Atom Free BODIPY-Related Dyes

Esther Rebollar [1], Jorge Bañuelos [2,*], Santiago de la Moya [3], Julien Eng [4], Thomas Penfold [4] and Inmaculada Garcia-Moreno [1,*]

[1] Departamento Química-Física de Materiales, Instituto de Química Física "Rocasolano", CSIC, Serrano 119, 28006 Madrid, Spain; e.rebollar@csic.es

[2] Departamento de Química Física, Universidad del País Vasco-EHU, Apartado 644, 48080 Bilbao, Spain

[3] Departamento Química Orgánica, Facultad de Ciencias Químicas, Universidad Complutense de Madrid, Ciudad Universitaria s/n, 28040 Madrid, Spain; santmoya@ucm.es

[4] Chemistry Department, School of Natural and Environmental Sciences, Newcastle University, Newcastle Upon-Tyne NE1 7RU, UK; julien.eng@newcastle.ac.uk (J.E.); tom.penfold@newcastle.ac.uk (T.P.)

* Correspondence: jorge.banuelos@ehu.es (J.B.); i.garcia-moreno@iqfr.csic.es (I.G.-M.)

Tables S2

Figures.. S4

Table S1. Photophysical properties of the commercial BODIPYs in diluted solutions (2 mM) of representative solvents.

	λ_{ab} (nm)	ε_{max} (10^4 $M^{-1}\cdot cm^{-1}$)	λ_{fl} (nm)	ϕ	τ (ns)
**PM546**					
c-hex	499.5	10.3	514.0	0.99	5.42
EtoAc	494.0	9.2	506.5	0.96	5.58
AcN	492.0	8.7	504.0	0.90	5.72
**PM556***					
AcN	506.0	8.5	531.5	0.84	5.13
H_2O	490.5	9.8	519.0	0.83	4.23
**PM567**					
c-hex	522.5	10.7	541.0	0.93	5.98
EtOAc	517.0	8.4	536.0	0.83	6.08
AcN	515.0	7.7	534.0	0.82	6.31
**PM597**					
c-hex	529.0	7.5	576.0	0.47	4.00
EtOAc	523.0	6.7	564.0	0.48	4.38
AcN	521.0	6.5	563.0	0.51	4.13
**PM650**					
c-hex	589.0	6.6	598.0	0.53	4.73
EtOAc	588.0	6.0	605.0	0.20	2.48
AcN	587.5	5.1	608.0	0.14	1.67

Absorption (λ_{ab}) and fluorescence (λ_{fl}) wavelength; molar absorption at the maximum (ε_{max}); fluorescence quantum yield (ϕ) and lifetime (τ).
c-hex: cyclohexane; EtOAc: ethyl acetate; AcN: acetonitrile
*not soluble in non-polar solvents

Table S2. LR-CC2 results of the low-lying excited states and oscillator strengths (in brackets) of PM546, PM567, PM597 and PM650 at the ground, S_1 and T_1 optimised geometries.

**GS Geom**	PM546	PM567	PM597	PM650
HOMO /eV	-7.14	-6.91	-6.89	-7.20
LUMO / eV	0.72	0.80	0.78	-0.15
GS / eV	0.00	0.00	0.00	0.00
T_1 / eV	2.05	2.00	1.97	1.31
T_2 / eV	3.19	3.03	2.99	2.02
T_3 / eV	3.36	3.26	3.23	2.89
S_1 / eV	2.85 (0.476)	2.73 (0.505)	2.69 (0.543)	2.12 (0.324)
S_2 / eV	3.68 (0.102)	3.57 (0.162)	3.51 (0.170)	3.32 (0.210)
S_3 / eV	3.90 (0.038)	3.73 (0.047)	3.65 (0.038)	3.53 (0.023)
**S_1 Geom**				
HOMO / eV	-6.96	-6.76	-6.74	-7.58
LUMO / eV	0.57	0.67	0.61	-0.59
GS / eV	0.59	0.42	0.45	0.62
T_1 / eV	2.63	2.37	2.31	2.20
T_2 / eV	2.83	2.65	2.51	2.36
T_3 / eV	3.73	3.48	3.40	3.40
S_1 / eV	2.95 (0.077)	2.62 (0.176)	2.48 (0.156)	2.39 (0.061)
S_2 / eV	3.35 (0.395)	3.22 (0.363)	3.05 (0.379)	3.02 (0.492)
S_3 / eV	4.40 (0.058)	4.09 (0.064)	3.96 (0.052)	3.82 (0.044)
**T_1 Geom**				
HOMO / eV	-7.52	-7.21	-7.23	-7.06
LUMO / eV	0.38	0.49	0.40	-0.34
GS / eV	0.08	0.03	0.03	0.34
T_1 / eV	1.92	1.85	1.84	1.77
T_2 / eV	3.29	3.05	2.98	3.04
T_3 / eV	3.31	3.22	3.21	3.29
S_1 / eV	2.88 (0.511)	2.71 (0.536)	2.65 (0.532)	2.50 (0.485)
S_2 / eV	3.66 (0.040)	3.48 (0.106)	3.36 (0.095)	3.03 (0.158)
S_3 /eV	3.87 (0.017)	3.68 (0.028)	3.59 (0.027)	3.23 (0.019)

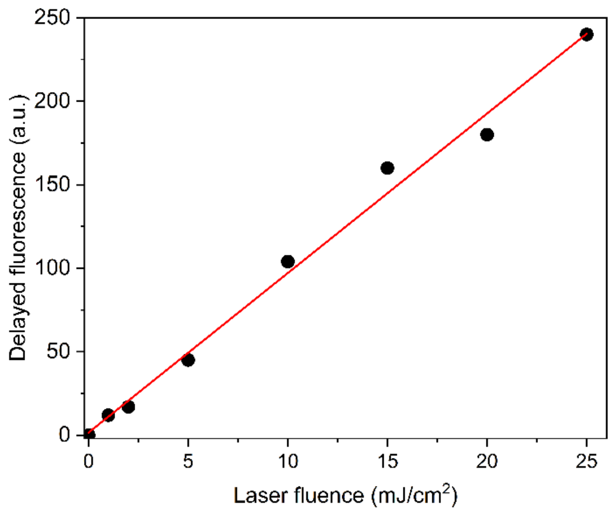

Figure S1. Intensity of the recorded delayed emission as a function of the laser pulse energy.

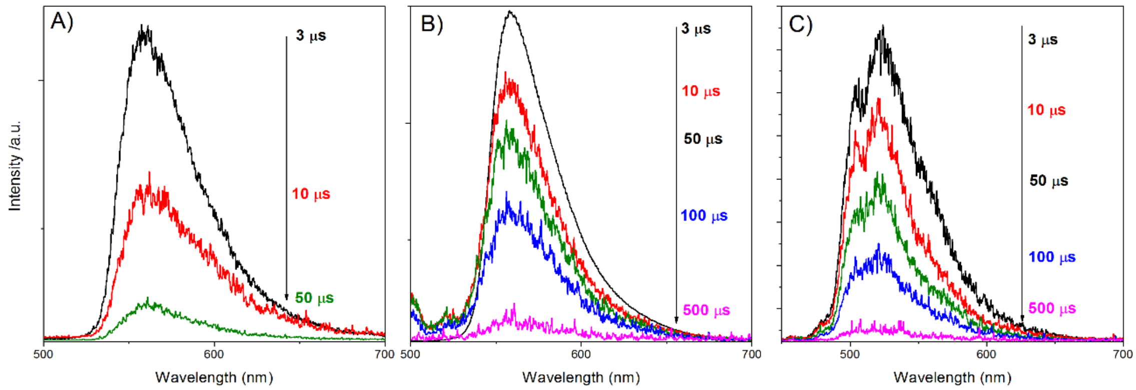

Figure S2. Time-dependent fluorescence emission spectra of *COO*-BODIPY **3** upon laser photo-excitation at 355 nm (A), and *N*-BODIPY **4** (B) and BOPHY **5** (C) after laser photo-excitation at 532 nm, in ethyl acetate aerated solution at room temperature.

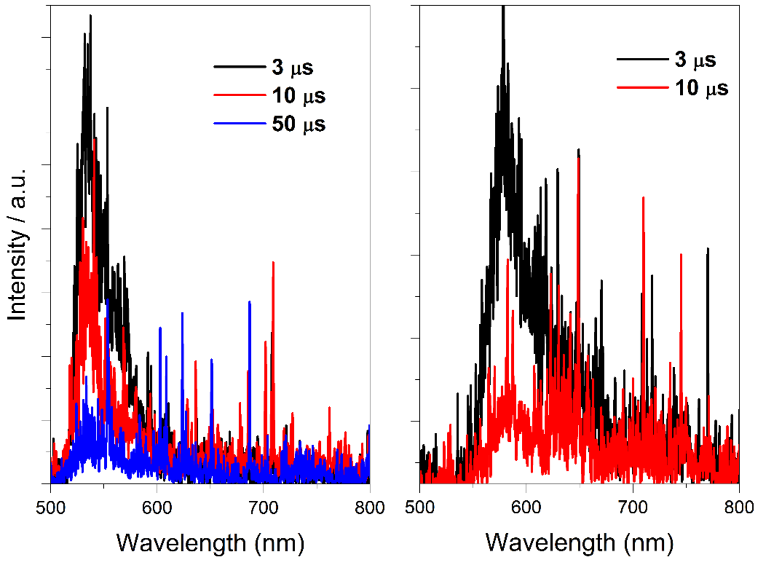

Figure S3. Time-resolved emission spectra at different delay times (in μs) after laser excitation at 355 nm of PM546 (left) and PM597 (right) in aerated ethyl acetate solution at room temperature.

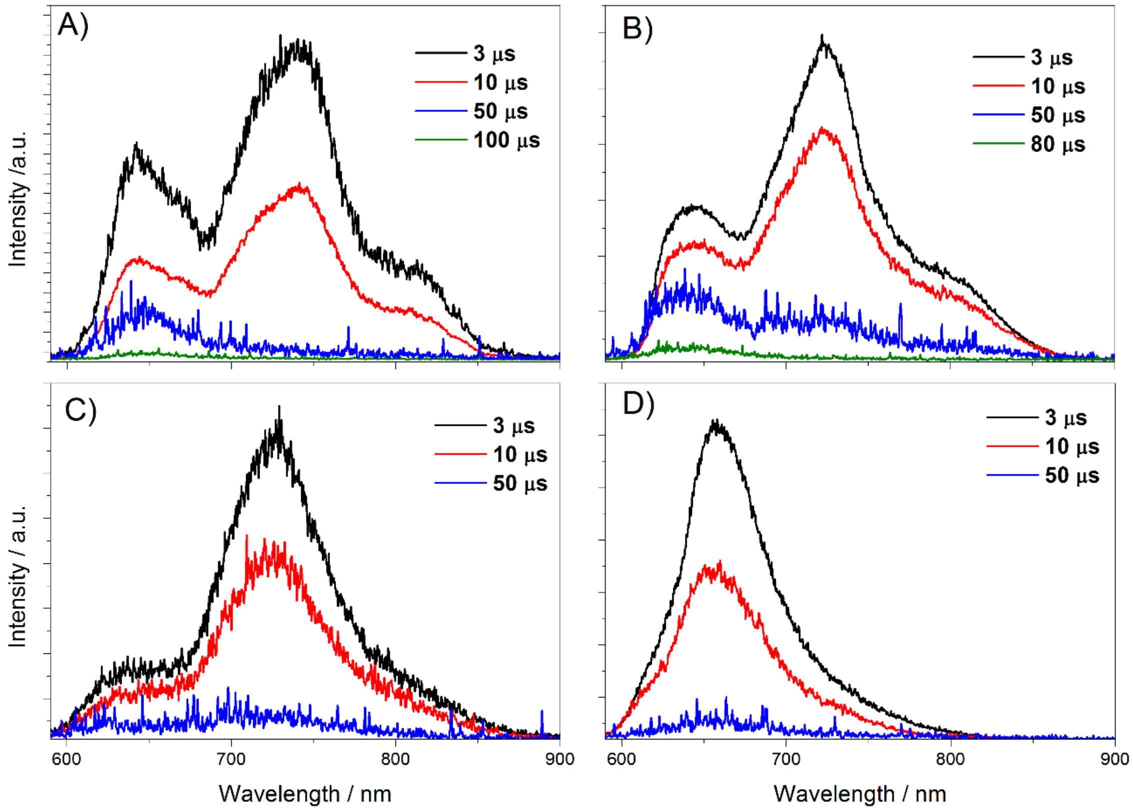

Figure S4. Comparison of the time-resolved emission spectra of PM650 in different solvents (toluene (A), ethyl acetate (B), acetonitrile (C) and DMSO (D)) upon laser photoexcitation at 532 nm. The measurements were carried out in aerated solutions at room temperature.
